# All‐Oral Shorter Treatment Regimens for Multidrug‐ and Rifampicin‐Resistant Tuberculosis: Evaluating Their Effectiveness, Safety, and Impact on the Quality of Life of Patients in Lao PDR

**DOI:** 10.1111/tmi.70041

**Published:** 2025-09-25

**Authors:** Vibol Iem, Sakhone Suthepmany, Vongkham Inthavong, Anousone Sisouvanh, Khamloun Choumlivong, Kyung Hyun Oh, Philipp du Cros, Fatimata Bintou Sall, Corinne S. Merle, Jacques Sebert, Donekham Inthavong

**Affiliations:** ^1^ Centre for Tuberculosis Research Liverpool School of Tropical Medicine Liverpool UK; ^2^ Office of the WHO Representative in Lao PDR Vientiane Lao PDR; ^3^ National Tuberculosis Control Center Vientiane Lao PDR; ^4^ Sethathirath Hospital Vientiane Lao PDR; ^5^ World Health Organization Regional Office for the Western Pacific Manila Philippines; ^6^ Burnet Institute Melbourne Australia; ^7^ Special Programme of Research and Training in Tropical Diseases (TDR) World Health Organization Geneva Switzerland

**Keywords:** all‐oral regimen, drug‐resistant tuberculosis, health‐related quality of life, programmatic implementation, rifampicin‐resistant TB, treatment outcomes

## Abstract

**Background:**

Drug‐resistant tuberculosis remains a major public health challenge in Lao PDR, with low second‐line treatment uptake and suboptimal outcomes. To improve effectiveness, safety, and tolerability, a shorter all‐oral regimen for multidrug‐ and rifampicin‐resistant tuberculosis (MDR/RR‐TB) was introduced under the TDR Short, all‐Oral Regimens for Rifampicin‐resistant Tuberculosis (ShORRT) initiative.

**Methods:**

A retrospective and prospective comparative cohort study was conducted across five drug‐resistant tuberculosis treatment centres from January 2020 to December 2023. Two programmatic cohorts were analysed during partially overlapping calendar periods: a standard injectable‐containing regimen cohort and an all‐oral regimen cohort. Outcomes were assessed at the end of treatment and 12 months post‐treatment. Safety was evaluated through adverse events, including serious adverse events and adverse events of special interest. Health‐related quality of life was measured using EQ‐5D‐5L and EQ‐VAS tools.

**Results:**

Among 126 participants, 65 received the all‐oral regimen and 61 the standard regimen. Treatment success was higher in the all‐oral group (90.8% vs. 80.3%), with lower mortality (7.5% vs. 16.4%) and fewer serious adverse events (12.3% vs. 19.7%). Anaemia was more common in the all‐oral group (46.2%), while hepatotoxicity and QTcF prolongation were more frequent in the standard group. Both groups showed improvements in health‐related quality of life, but greater recovery in mobility, daily activities, and anxiety reduction was observed in the all‐oral group. Between group differences did not reach statistical significance. No cases of tuberculosis recurrence were reported at 12‐month follow‐up in either group.

**Conclusion:**

In this programmatic setting, the all‐oral, bedaquiline and linezolid‐based regimen demonstrated high effectiveness and acceptable safety. Non‐significant trends favoured the all‐oral regimen for treatment success, mortality, and quality of life, consistent with but not definitive for improved outcomes. These findings support the transition to all‐oral regimens as the preferred approach for drug‐resistant tuberculosis care, while acknowledging the observational design and limited power.

**Abbreviations**
AE: Adverse Event
AESI: Adverse Event of Special Interest
ALT: Alanine Aminotransferase (SGPT)
AST: Aspartate Aminotransferase (SGOT)
Bdq: Bedaquiline
BMI: Body Mass Index
Cfz: Clofazimine
CI: Confidence Interval
DAIDS: Division of AIDS (grading criteria)
DR‐TB: Drug‐Resistant Tuberculosis
E: Ethambutol
EQ‐5D: EuroQol 5‐Dimension Questionnaire
EQ‐VAS: EuroQol Visual Analogue Scale
Fq: Fluoroquinolone
H: Isoniazid
HIV: Human Immunodeficiency Virus
HRQoL: Health‐Related Quality of Life
Lfx: Levofloxacin
Lzd: Linezolid
Mfx: Moxifloxacin
Pto: Prothionamide
RR‐TB: Rifampicin‐Resistant Tuberculosis
SAE: Serious Adverse Event
ShORRT: Short, all‐Oral Regimens for Rifampicin‐Resistant Tuberculosis
TB: Tuberculosis
TDR: Special Programme for Research and Training in Tropical Diseases (WHO)
WHO: World Health Organization
Z: Pyrazinamide

## 1 Introduction

Although tuberculosis (TB) is both preventable and treatable, it likely regained its position in 2023 as the world's leading cause of death from a single infectious agent, after being temporarily surpassed by Coronavirus disease (COVID‐19) over the past 3 years [1]. Of particular concern is the growing burden of drug‐resistant TB (DR‐TB), which accounts for one‐third of all antimicrobial resistance‐related deaths, killing nearly 200,000 people each year [1]. Rifampicin‐resistant TB (RR‐TB) and multidrug‐resistant TB (MDR‐TB), resistant to both rifampicin and isoniazid (the two key first‐line TB drugs), pose a serious threat to global health security, with far‐reaching implications for individuals, communities, and healthcare systems [2]. In 2023, only 44% of people estimated to have multidrug‐ or rifampicin‐resistant tuberculosis (MDR/RR‐TB) globally were diagnosed and treated, leaving the majority undiagnosed and continuing to fuel transmission [1]. Among those who did access second‐line treatment, just 68% achieved treatment success, underscoring the limited effectiveness of current regimens [1]. Many of these regimens are lengthy, toxic, and complex, with high pill burdens and prolonged duration contributing to poor adherence and unfavourable outcomes [3]. Care is further complicated by comorbidities such as HIV, diabetes, and liver disease, which increase the risk of adverse drug reactions and treatment interruptions [4]. Although shorter, all‐oral regimens offer promise in improving outcomes and reducing toxicity [5, 6, 7], their scale‐up remains constrained by diagnostic delays, laboratory limitations, and insufficient capacity at peripheral health facilities [8].

In the Lao People's Democratic Republic (Lao PDR), TB remains a pressing public health issue despite progress over the past two decades, with an estimated 10,000 (132 [81–197]/100,000) new and relapse TB cases annually, of which only around 9000 are reported [1]. Treatment success for new and relapse TB cases reached 88% in 2022, but death (6.4%) and loss to follow‐up (4.6%) remain concerns. The national anti‐TB drug resistance survey in 2017 provided estimates of rifampicin resistance of 1.2% (0.5%–2.0%) among new cases and 4.1% (0.9%–6%) among previously treated cases [9]. Based on the rates of rifampicin resistance detected through the routine programme, the estimates of MDR/RR‐TB have been reduced to 0.67% (0.49–0.87) among new cases and increased to 11% (7.9–15) among previously treated cases. DR‐TB remains challenging, with only 51% (2022) and 47% (2023) of estimated MDR/RR‐TB cases starting second‐line treatment. Among those diagnosed in 2022, treatment success was 60%, while 9.8% refused treatment and 7.8% died before starting it. Among patients who initiated treatment, 12.5% died during therapy and 10% were lost to follow‐up (Lao NTP unpublished data).

In 2020, to improve the programmatic management of drug‐resistant TB, Lao PDR transitioned to all‐oral treatment using the ShORRT (Short, all‐Oral Regimens for Rifampicin‐resistant Tuberculosis) operational research package [10], in line with the 2020 WHO consolidated guidelines on tuberculosis. Module 4: treatment—drug‐resistant tuberculosis treatment. During this national transition, both the legacy standard regimen and all‐oral regimen were delivered under routine programmatic conditions in partially overlapping periods, enabling comparative cohort analysis.

This study aims to assess the safety, effectiveness, and impact on health‐related quality of life (HRQoL) of an all‐oral shorter MDR/RR‐TB treatment regimen under programmatic conditions in Lao PDR.

## 2 Materials and Methods

### 2.1 Study Design

**FIGURE 1:**
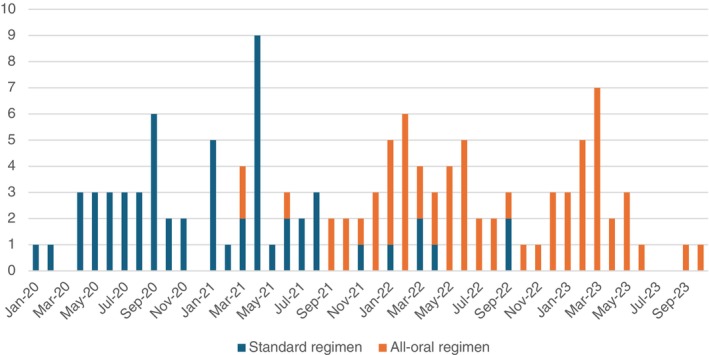
Monthly number of patients initiated by regimen type showing overlapping enrolment of the standard regimen cohort and all oral regimen cohort during the transition period.

This retrospective and prospective comparative cohort study included two distinct programmatic cohorts in Lao PDR. It was not a randomised or stepwise implementation study. The study was conducted as part of operational research embedded within the national TB programme activities, under routine programmatic standards of care. From January 2020 to March 2021, all patients were treated with the standard regimen (injectable DR‐TB treatment regimen). Following approval, a phased transition to the all‐oral treatment regimen began in March 2021 and continued gradually until full adoption in September 2022, when all patients were initiated on the all‐oral regimen, as shown in Figure 1. Both patients' cohorts were followed up to 12 months after the end of the treatment. This approach allowed for a comprehensive monitoring of patient outcomes across both treatment and follow‐up periods, with semi‐concurrent comparison between the two cohorts. Beyond the regimen change itself, programmatic standards of care, clinical monitoring schedules, and data collection procedures were consistent across cohorts. As such, this analysis is a comparative observational study, reflecting real‐world conditions rather than a controlled experimental assignment.

Only a brief overview of the ShORRT protocol is provided here for context. The full schedule of assessments, dosing, and monitoring procedures is presented in the Supporting Information: Appendix.

### 2.2 Protocol Adaptations and Laos Specific Implementation

We followed the ShORRT operational research package but introduced context‐specific adaptations. First, enrolment used two cohorts delivered during overlapping periods to reflect the national transition, rather than a stepwise switch at a single timepoint. Second, all oral regimens were prioritised for fluoroquinolone‐sensitive disease, with standard regimens reserved for selected cases during the transition. Third, ECG monitoring was performed at baseline, weeks one and two, then monthly, with additional checks if clinically indicated; linezolid dose reductions or temporary interruptions were applied for suspected myelosuppression or neuropathy. Fourth, pharmacovigilance used the national aDSM procedures, with causality assessed by site clinicians and reviewed centrally. Fifth, health‐related quality of life was measured with EQUATION 5D 5 L and EQ VAS at baseline and end of treatment, and again at 12 months after treatment to capture post‐tuberculosis recovery. These choices reflect routine programmatic practice in Laos and are described in full in Supporting Information: Appendix.

### 2.3 Study Sites

This study was conducted at the five designated DR‐TB treatment centres in Lao PDR: Sethathirath Hospital in Vientiane Capital, and the provincial hospitals in Champasack, Khammouan, Louang Prabang, and Savannakhet provinces. The programme management of drug‐resistant TB (PMDT) began in 2011 in Lao PDR with the opening of the DR‐TB treatment centre in Sethathirath central hospital, followed by the gradual expansion to the four provincial hospitals in the years that followed. These centres are part of the National TB Programme (NTP) and have consistently delivered care in accordance with the latest WHO recommendations for the PMDT.

Since 2011, Lao PDR has adopted all WHO‐endorsed regimens as they became available. Initial treatment relied on long, injectable‐based regimens with modest success rates (averaging 60%). In 2013, a shorter 9‐month regimen was introduced under operational research, achieving success rates above 80% among enrolled patients from 2015 onwards. As resistance patterns evolved, the country incorporated newer, individualised regimens using bedaquiline, delamanid, and linezolid. In 2020, Lao PDR transitioned to all‐oral regimens through the TDR ShORRT operational research package, marking a major step towards safer, more tolerable DR‐TB care.

In Lao PDR, all TB diagnostic services, medications, and clinical care are provided free of charge through the NTP. DR‐TB treatment is delivered through a patient‐centred approach, with an emphasis on education, counselling, and support throughout the treatment journey. Patients typically begin treatment in one of the five DR‐TB treatment centres and may later transition to ambulatory care once deemed non‐contagious. Treatment is supported through directly observed therapy (DOT), either facility‐based or conducted in the community by trained health workers, village volunteers, or family members.

To promote adherence and improve outcomes, a structured support package was available at all sites. The minimum package consisted of monthly counselling, close clinical monitoring, transport reimbursement, and nutritional supplements. Depending on local resources, additional support included food packages and community follow‐up visits by health workers. Infection prevention counselling, family education, and psychosocial support were integral components of care.

### 2.4 Study Period

The study comprised two cohorts enrolled in overlapping calendar periods. The standard regimen cohort included individuals enrolled between 01 January 2020 and 31 December 2020, with retrospective data capture for early months and prospective capture thereafter. The all oral regimen cohort included individuals enrolled between 01 January 2021 and 31 December 2023, with prospective data capture throughout. All participants underwent a 9‐ to 12‐month treatment period, followed by a 12‐month post‐treatment follow‐up to assess long‐term outcomes.

### 2.5 Study Population

Inclusion criteria were age ≥ 18 years, bacteriologically confirmed rifampicin resistance (identified using Xpert MTB/RIF or Xpert MTB/RIF Ultra, with confirmatory phenotypic DST performed when feasible), and willingness and ability to provide informed consent. Exclusion criteria were severe comorbidities defined as conditions likely to compromise treatment safety or adherence, such as advanced HIV disease with CD4 < 200 cells/μL, uncontrolled diabetes mellitus with complications, decompensated liver disease, chronic renal failure requiring dialysis, or severe malnutrition (BMI < 14 kg/m^2^ or clinical signs of organ dysfunction). Other exclusion criteria were known allergies or contraindications to any drugs in the study regimen, refusal to participate, inability to take oral medication, or a QTcF interval ≥ 500 msec at baseline that could not be corrected with medical management.

### 2.6 Diagnostic Procedures

Diagnosis of MDR/RR‐TB was conducted using the Xpert MTB/RIF and Xpert MTB/RIF Ultra assays (Cepheid, Sunnyvale, USA). Additional phenotypic drug susceptibility testing (DST) for isoniazid and second‐line anti‐TB drugs was performed at the National TB Reference Laboratory (Lao NRL) in Vientiane Capital, using the absolute concentration method. The Lao NRL maintains high technical standards through active participation in an External Quality Assessment (EQA) programme provided by the Korean Institute of Tuberculosis (KIT). As the designated Supranational Reference Laboratory for Lao PDR, KIT, also a WHO Collaborating Centre and a member of the WHO Supranational TB Reference Laboratory Network [11], supports the Lao NRL across all laboratory techniques, with a particular focus on DST. The DST EQA is conducted and renewed annually, ensuring the laboratory's sustained compliance with international standards and the reliability of diagnostic results. The Lao NRL has consistently achieved satisfactory performance in this programme, supporting the reliability of diagnostic results.

### 2.7 Treatment Regimens

Two programmatic regimens were used according to national adoption of the ShORRT package and WHO guidance. The standard regimen included an injectable aminoglycoside (amikacin), high‐dose isoniazid, protionamide, clofazimine, pyrazinamide, moxifloxacin, and ethambutol. The all‐oral regimen replaced the injectable and companion drugs with bedaquiline, linezolid, and levofloxacin, while retaining clofazimine and pyrazinamide. Drug lists, dosing by weight band, and planned durations are provided in Tables S1 and S2. Dosing adjustments and stopping rules for linezolid and high‐dose isoniazid followed national pharmacovigilance procedures.

### 2.8 Study Outcomes

The primary outcomes of this study were treatment effectiveness, treatment safety, and health‐related quality of life (HRQoL) [12]. Treatment effectiveness was defined as the proportion of participants who achieved treatment success, including cure or treatment completion without microbiological evidence of failure, assessed at the end of treatment and at 12 months post‐treatment, in accordance with WHO definitions [13]. Treatment safety was assessed based on the occurrence, incidence, and severity of serious adverse events (SAEs, defined as events leading to death, hospitalisation, life‐threatening conditions, or permanent disability) and adverse events of special interest (AESIs), including anaemia, leukopenia, thrombocytopenia, elevated liver enzymes, QTcF prolongation, peripheral neuropathy, psychiatric effects (including anxiety and depression), and ototoxicity. AESIs were graded according to the Division of AIDS (DAIDS) criteria [14]. HRQoL was evaluated using the EQ‐5D‐5L questionnaire [15] and the EuroQol Visual Analogue Scale (EQ‐VAS) [16], administered at baseline and at the end of treatment.

Secondary outcomes included treatment failure, defined as persistent positive cultures or clinical deterioration requiring treatment modification; mortality, recorded both during treatment and post‐treatment follow‐up; and loss to follow‐up, defined as the failure to attend scheduled visits for at least two consecutive months. Additional secondary outcomes included time to culture conversion, measured from treatment initiation to the first of two consecutive negative cultures obtained at least 30 days apart, and treatment adherence, assessed as the proportion of participants completing treatment without exceeding predefined missed‐dose thresholds.

### 2.9 Monitoring and Follow‐Up

To assess these outcomes, patients were monitored monthly for clinical response, drug‐related AEs, and treatment adherence. Sputum samples were collected monthly for smear microscopy and culture to monitor bacteriological response. The AESIs were systematically recorded and graded according to the DAIDS Table for Grading the Severity of Adult and Paediatric Adverse Events [14].

Follow‐up at six and 12 months after treatment included clinical evaluation, symptom screening, and bacteriological testing to detect recurrence, conducted in line with WHO recommendations. For participants who missed appointments, study staff followed up actively to support return to care. The same visit schedules, investigations, and pharmacovigilance procedures were applied in both cohorts under routine standards of care. The full visit schedule is presented in Table S3.

### 2.10 Data Collection

Data were collected using standardised case report forms and entered into the REDCap electronic data capture tool hosted at WHO (Geneva) [17]. Socio‐demographic information, clinical characteristics, treatment regimen details, treatment outcomes, and laboratory data were collected using both routinely used forms and study‐specific forms. Treatment outcomes were classified according to WHO definitions, including cure, treatment completion, treatment failure, death, and loss to follow‐up [13]. AEs and clinical data were recorded in patients' medical records and an adverse drug event monitoring system (aDSM). HQoL was assessed at baseline and at the end of the treatment using the EQ‐5D‐5L as described in the study by Rodriguez et al. [18]. Data completeness was actively promoted through close follow‐up of missed visits and cross‐checking between clinical records and study‐specific forms, ensuring near‐complete outcome ascertainment.

### 2.11 Data Processing and Analysis

Group comparisons were conducted as observational cohort comparisons; the study did not involve random allocation or a stepwise implementation design. Basic descriptive statistics and cross‐tabulations were generated to describe the patient population characteristics, TB care model, demographics, socio‐economic position, and TB treatment information (treatment adherence, AEs, and treatment outcomes).

Continuous variables were expressed as means with standard deviations or medians with first and third quartiles, while categorical variables were expressed as absolute frequencies and percentages. Comparisons between groups were made using Pearson's chi‐squared or Fisher's exact tests for categorical variables and t‐tests or Wilcoxon rank‐sum tests for continuous variables, as appropriate. Comparisons of treatment effectiveness and safety between the two regimens were assessed using a Poisson regression model, with results presented as risk ratios (RR) and their corresponding 95% confidence intervals (CI). The difference in EQ‐VAS scores between baseline and the end of treatment was compared between the two groups using a linear regression model, with results expressed as the beta coefficient and its corresponding 95% confidence interval. Baseline covariates with a *p* ≤ 0.10 were included in the model for adjustment. All statistical tests were performed with a 5% significance level (*α* = 0.05). Data were managed using R version 4.2.3 [19] for analysis, with real‐time monitoring for completeness and accuracy.

The datasets used and/or analysed during the current study are available from the corresponding author on reasonable request for guideline development and systematic reviews.

## 3 Ethical Considerations

Ethical approval was granted by the WHO Research Ethics Review Committee (Protocol ID: ERC.0003305) and the National Ethics Committee for Health Research in Lao PDR (Protocol ID: 2020.82.MP). Informed consent was obtained from all participants, and the study was conducted according to Good Clinical Practice [20]. For the prospective phase of the study, written informed consent was obtained prior to enrolment. Participants were informed of the study objectives, procedures, risks, and their rights, including the right to withdraw at any time without affecting their access to care. For the retrospective component involving medical record review, a waiver of informed consent was granted, as the data were de‐identified and posed minimal risk to participants.

## 4 Results

**FIGURE 2:**
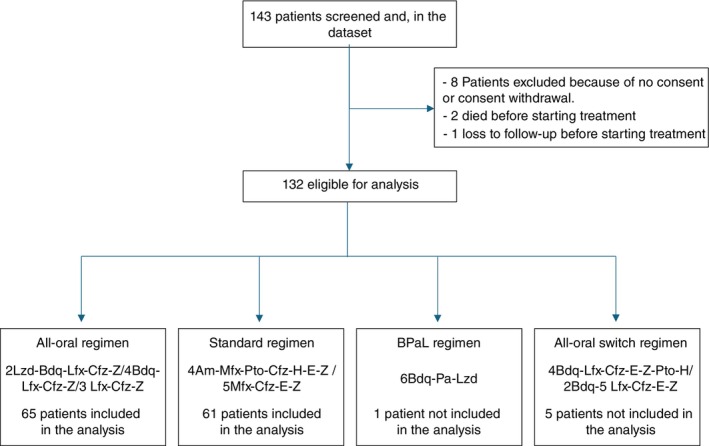
Study flowchart.

We analysed outcomes from two programmatic cohorts delivered under routine conditions during partially overlapping (semi‐concurrent) calendar periods in Lao PDR, within the framework of the ShORRT package but with local adaptations described in the Supporting Information: Appendix. A total of 143 individuals were screened, with 132 eligible for analysis. Eleven participants were excluded due to lack of consent (*n* = 8), death prior to treatment initiation (*n* = 2), and loss to follow‐up before treatment (*n* = 1). Among those included, 65 received the all‐oral regimen, while 61 were assigned to the standard regimen. Additionally, one participant received the BPaL regimen, and five participants received an all‐oral switch regimen. These two latter (BPaL regimen and all‐oral switch regimen) are not included in this analysis (Figure 2).

Table 1 presents the baseline characteristics of study participants by treatment group. The distribution of demographic and clinical variables was broadly comparable between the all‐oral and standard regimen groups. Participants in both groups were of similar age, with a median of 49 years (IQR 37–57) in the all‐oral group and 53 years (IQR 38–62) in the standard group (*p* = 0.3), and most were male (64.6% vs. 70.5%, *p* = 0.4).

**TABLE 1:** Baseline characteristics of study participants.

	All‐oral regimen *N* = 65^a^	Standard regimen *N* = 61^a^	*p* ^b^
Age (years)			0.3
Mean (SD)	47 (14)	49 (15)	
Median (IQR)	49 (37–57)	53 (38–62)	
Age groups (years)			> 0.9
15–24	5 (7.7%)	5 (8.2%)	
25–44	20 (30.8%)	20 (32.8%)	
≥ 45	40 (61.5%)	36 (59.0%)	
Sex			0.4
Male	42 (64.6%)	43 (70.5%)	
Female	23 (35.4%)	18 (29.5%)	
Type of patient			0.4
New TB case	23 (35.4%)	21 (34.4%)	
Relapse	37 (56.9%)	33 (54.1%)	
Treatment after LTFU	2 (3.1%)	0 (0.0%)	
Treatment after failure	3 (4.6%)	6 (9.8%)	
Other previously treated patients	0 (0.0%)	1 (1.6%)	
Disease site			0.2
Pulmonary	64 (98.5%)	57 (93.4%)	
Extra‐pulmonary	1 (1.5%)	4 (6.6%)	
History with TB drugs			> 0.9
Previously treated only with first line drugs	42 (64.6%)	40 (65.6%)	
Clinical evaluation			
BMI in kg/m^2^ (median (IQR))	18.00 (16.5, 19.9)	18.20 (16.5, 20.0)	> 0.9
Comorbidities			
Existing neuropathy	4 (6.7%)	6 (11.3%)	0.022
HIV Status	1 (1.5%)	6 (9.8%)	< 0.001
Hepatitis B	7 (12.7%)	5 (8.2%)	0.2
Hepatitis C	4 (7.4%)	4 (6.6%)	0.2
Biological parameters (median (IQR))			
Haemoglobin (g/dL)	12.1 (10.3, 13.3)	12.8 (11.5, 14.1)	0.2
Platelet (1000/μl)	357 (284, 449)	273 (196, 350)	< 0.001
White blood count (1000/μl)	10.4 (8.0, 12.8)	9.1 (7.0, 10.8)	0.044
Glucose (mg/dL)	0.94 (0.8, 1.3)	0.87 (0.7, 1.0)	0.3
AST/SOPT (U/L)	25 (18, 35)	26 (18, 38)	> 0.9
Potassium (mmol/L)	4.10 (3.7, 4.4)	3.8 (3.5, 4.1)	0.022
Creatinine (mg/dL)	0.9 (0.8, 1.1)	0.8 (0.7, 1.0)	0.2
Chest X‐Ray			
Cavity	54 (83.1%)	36 (59.0%)	0.002
Extend of the disease^c^			0.3
A (< 25%)	22 (39.3%)	11 (22.4%)	
B (25%–49%)	28 (50.0%)	32 (65.3%)	
C (> 50%)	5 (8.9%)	5 (10.2%)	
Bacteriological characteristics			
Positive sputum smear	13 (22.1%)	19 (37.3%)	0.024
Positive sputum culture	31 (51.7%)	19 (35.2%)	0.11
Isoniazid resistant	24 (52.2%)	26 (55.3%)	0.5
Fluoroquinolone sensitivity			0.2
Sensitive	52 (81.2%)	42 (70.0%)	
Unknown	14 (18.8%)	19 (30.0%)	
Disability assessment			0.6
Breathless with strenuous exercise	14 (33.3%)	11 (29.7%)	
Short breath	19 (45.2%)	17 (45.9%)	
Walk slower	6 (14.3%)	3 (8.1%)	
Stop for breath	2 (4.8%)	2 (5.4%)	
Breathless	1 (2.4%)	4 (10.8%)	

^a^ *n* (%).

^b^ Wilcoxon rank sum test; Fisher's exact test; Pearson's Chi‐squared test.

^c^ Extent of disease was assessed based on the degree of radiographic involvement observed on chest X‐ray at baseline.

The groups were similar with respect to key clinical characteristics, including BMI, type of TB case, and history of prior treatment. Several notable differences were observed: HIV co‐infection and pre‐existing neuropathy were more common in the standard regimen group, while cavitary disease on chest X‐ray was more frequent in the all‐oral group. A higher proportion of participants in the standard group were also smear‐positive at baseline. Other differences in laboratory parameters (e.g., platelet counts, white blood cell counts, serum potassium) reached statistical significance but are unlikely to be clinically meaningful. Overall, the two cohorts were reasonably balanced, and baseline differences were accounted for in adjusted analyses.

Table 2 shows that treatment outcomes at the end of follow‐up were generally favourable in both groups. In the all‐oral regimen group, 90.8% of participants achieved a favourable outcome (defined as cure or treatment completion without evidence of failure or relapse) compared to 80.3% in the standard regimen group. Although the difference was not statistically significant (IRR 1.1; 95% CI: 0.7–1.8; *p* = 0.578), treatment success was 90.8% in the all‐oral group compared to 80.3% in the standard group.

**TABLE 2:** End of treatment and follow up outcomes' summary.

	All‐oral regimen *N* = 65^a^	Standard regimen *N* = 61^a^	IRR^b^ (95% CI)	*p*
Outcome 12 months after the end of the treatment			1.1 (0.7–1.8)	0.578
**Favourable**	**59 (90.8%)**	**49 (80.3%)**		
Cured	40 (61.5%)	43 (70.5%)		
Treatment completed	19 (29.3%)	6 (9.8%)		
**Unfavourable**	**6 (9.2%)**	**12 (19.7%)**		
Died during treatment	5 (7.5%)	10 (16.4%)		
Treatment failure	0 (0.0%)	0 (0.0%)		
Loss of follow‐up	1 (1.5%)	2 (3.3%)		
Recurrent episode	0 (0.0%)	0 (0.0%)		
**Treatment adherence**	**63 (96.9%)**	**60 (98.4%)**		

^a^ *n* (%).

^b^ Incidence rate ratio; CI, confidence interval.

Within the all‐oral group, 61.5% of participants were classified as cured and 29.3% had completed treatment without confirmed bacteriological cure, compared to 70.5% and 9.8%, respectively, in the standard regimen group.

Unfavourable outcomes were less frequent with the all‐oral regimen (9.2% vs. 19.7%), primarily driven by a lower proportion of deaths during treatment (7.5% vs. 16.4%). Loss to follow‐up was also slightly lower in the all‐oral group (1.5% vs. 3.3%). Importantly, no cases of treatment failure or recurrent TB were reported in either group. Additionally, no participants were classified as “not assessable” at final evaluation.

Treatment adherence was high in both groups, with 96.9% of participants in the all‐oral group and 98.4% in the standard group completing treatment within predefined adherence thresholds.

Table 3 summarises the occurrence and characteristics of serious adverse events (SAEs) and adverse events of special interest (AESIs). A lower proportion of participants in the all‐oral regimen group experienced at least one SAE compared to the standard regimen group (12.3% vs. 19.7%; *p* = 0.155). A lower proportion of participants in the all‐oral regimen group experienced at least one SAE compared to the standard regimen group (12.3% vs. 19.7%; IRR 0.5, 95% CI: 0.2–1.3; *p* = 0.155).

**TABLE 3:** Serious adverse events, proportion, severity, and frequency per patient.

	All‐oral regimen	Standard regimen	IRR^b^ ^s^ (95% CI)	*p*
Serious adverse events (SAEs)	*N* = 65^a^	*N* = 61^a^		
Had at least one serious adverse event	8 (12.3%)	12 (19.7%)	0.5 (0.2–1.3)	0.155
Type of SAE	*N* = 9	*N* = 13		
Death	5 (55.6%)	10 (76.9%)		
Life‐threatening experience	2 (22.2%)	2 (15.4%)		
Any hospitalisation or prolongation of hospitalisation	0 (0.0%)	1 (7.7%)		
Persistently or significantly disabling event	1 (11.1%)	0 (0.0%)		
Other medically important event	1 (11.1%)	0 (0.0%)		
SAE related to use of medication				
Related	2 (22.2%)	3 (23.1%)		
Not related	4 (44.4%)	10 (76.9%)		
Insufficient data to assess	3 (33.3%)	0 (0.0%)		
SAE requiring change of regimen	8 (88.9%)	12 (92.3%)		
Adverse events of special interest				
Proportion of patients experiencing each AESI	*N* = 65^a^	*N* = 61^a^		
**Anaemia**	**30 (46.2%)**	**14 (23.0%)**		
Thrombocytopenia	9 (13.8%)	8 (13.1%)		
Leukopenia	0 (0.0%)	1 (1.6%)		
Elevated liver enzymes	38 (58.5%)	45 (73.8%)		
QTcF prolongation	37 (56.9%)	40 (65.6%)		
Optic neuritis	0 (0.0%)	1 (1.6%)		
Type of AESI and their grading (total events)	*N* = 209^a^	*N* = 197^a^		
Anaemia	42 (20.1%)	17 (8.6%)		
Grade 1	20 (47.6%)	12 (70.6%)		
Grade 2	7 (16.7%)	1 (5.9%)		
Grade 3	8 (19.0%)	2 (11.8%)		
Grade 4	7 (16.7%)	2 (11.8%)		
**Thrombopenia**	**9 (4.3%)**	**9 (4.6%)**		
Grade 1	4 (44.4%)	4 (44.4%)		
Grade 2	2 (22.2%)	2 (22.2%)		
Grade 3	0 (0.0%)	1 (11.1%)		
Grade 4	3 (33.3%)	2 (22.2%)		
**Leukopenia**	**0 (0.0%)**	**1 (0.5%)**		
Grade 1	0 (0.0%)	1 (100.0%)		
**Prolonged QTcF**	**86 (41.1%)**	**72 (36.5%)**		
Grade 1	22 (25.6%)	22 (30.6%)		
Grade 2	43 (50.0%)	35 (48.6%)		
Grade 3	21 (24.4%)	15 (20.8%)		
**Elevated liver enzymes**	**72 (34.4%)**	**97 (49.2%)**		
Grade 1	38 (52.8%)	43 (44.3%)		
Grade 2	24 (33.3%)	41 (42.3%)		
Grade 3	7 (9.7%)	11 (11.3%)		
Grade 4	3 (4.2%)	2 (2.1%)		
**Optic neuritis**	**0 (0.0%)**	**1 (0.5%)**		
Grade 2	0 (0.0%)	1 (100.0%)		
Number of episodes and type of AESI per patient	*N* = 51	*N* = 51		
Mean number of episodes of AESI per patient (SD)	4 (3)	4 (2)		
Mean number of different types of AESI per patient (SD)	2 (1)	2 (1)		

*Note*: Percentages in the upper section of the table refer to the proportion of patients who experienced each AESI. Percentages in the lower section refer to the proportion of all AESI episodes by type and severity. *p*‐values and confidence intervals are reported only for the primary outcome comparison (favourable versus unfavourable outcome at 12 months). The subcategories (such as cure, treatment completion, death, loss to follow‐up) are presented descriptively to show the distribution of events within each outcome group, but no inferential testing was performed for these individual rows. This approach avoids problems of multiple comparisons, which could lead to spurious findings, and reflects the fact that the study was not powered to detect statistically significant differences in each specific sub‐outcome (in bold).

Abbreviations: CI, Confidence Interval; SD, Standard Deviation.

^a^ *n* (%).

^b^ Incidence rate ratio.

Among reported SAEs, death was the most frequent event, accounting for 55.6% (95% CI: 21.2–86.3) of SAEs in the all‐oral group and 76.9% (95% CI: 46.2–95.0) in the standard group, with no statistically significant difference between groups (*p* = 0.37). Life‐threatening events and other medically important events were less common, and hospitalisations were reported only in the standard regimen group. Persistently disabling events were rare, occurring in one participant from the all‐oral group. Most SAEs were deemed unrelated to treatment, although causality could not be assessed for 33.3% of SAEs in the all‐oral group due to incomplete clinical documentation or insufficient information for formal attribution. Nearly all SAEs requiring treatment modification resulted in changes in therapy across both groups.

Regarding AESIs, anaemia was the most commonly reported event in the all‐oral regimen group (46.2% of participants), whereas elevated liver enzymes (58.5% vs. 73.8%) and QTcF prolongation (56.9% vs. 65.6%) were more frequent in the standard regimen group. Rates of thrombocytopenia were comparable between groups (approximately 13%), while leukopenia and optic neuritis were rare.

The average number of AESI episodes per participant was similar across groups (mean 4), although the all‐oral group exhibited a slightly wider range of episodes (1–11 vs. 1–9). The mean number of different AESI types experienced per participant was two in both groups.

In terms of severity, most AESIs were graded as mild or moderate. A slightly higher proportion of grade 4 events was observed in the all‐oral group (6.2% vs. 3.0%), contributed primarily by haematological toxicities such as severe anaemia and thrombocytopenia. In contrast, elevated liver enzymes and QTcF prolongation were more frequently graded as moderate to severe among participants in the standard regimen group.

Table 4 presents changes in health‐related quality of life (HRQoL) outcomes, assessed by the EQ‐VAS and EQ‐5D instruments. Participants' self‐reported health status (EQ‐VAS) improved over time in both treatment groups. At baseline, the mean EQ‐VAS score was slightly higher in the all‐oral regimen group (80.0, SD: 10.1) compared to the standard regimen group (78.3, SD: 10.0), indicating similar perceived health at treatment initiation. By 12 months post‐treatment, mean EQ‐VAS scores increased to 86.0 (SD: 7.0) and 84.8 (SD: 6.3), respectively, reflecting meaningful recovery in perceived health status across both groups.

**TABLE 4:** Health‐related quality of life outcomes: EQ‐VAS and EQ‐5D changes over time.

	All‐oral regimen	Standard regimen	Beta (95% CI)	*p*
EQ‐VAS score				
Baseline	*N* = 61	*N* = 45		
Mean (SD)	80.0 (10.1)	78.3 (10.0)		
12 months after the end of treatment	*N* = 59	*N* = 46		
Mean (SD)	86.0 (7.0)	84.8 (6.3)		
Score difference baseline and end of follow‐up	*N* = 55	*N* = 39		
Mean (SD)	5.2 (9.6)	6.2 (9.2)	0.11 (−3.8–4.0)	> 0.9
Change in EQ‐5D between baseline and end of follow‐up	*N* = 57	*N* = 39		
Mobility				
No problems—no change	9 (16%)	7 (18%)		
Any problems—no change	13 (23%)	15 (38%)		
Better	30 (53%)	15 (38%)		
Worse	5 (8.8%)	2 (5.1%)		
Self‐care				
No problems—no change	8 (14%)	4 (10%)		
Any problems—no change	12 (21%)	12 (31%)		
Better	28 (49%)	16 (41%)		
Worse	9 (16%)	7 (18%)		
Usual activities				
No problems—no change	12 (21%)	4 (10%)		
Any problems—no change	13 (23%)	11 (28%)		
Better	23 (40%)	20 (51%)		
Worse	9 (16%)	4 (10%)		
Pain/discomfort				
No problems—no change	8 (14%)	4 (10%)		
Any problems—no change	16 (28%)	12 (31%)		
Better	25 (44%)	21 (54%)		
Worse	8 (14%)	2 (5.1%)		
Anxiety/depression				
No problems—no change	11 (19%)	8 (22%)		
Any problems—no change	16 (28%)	10 (26%)		
Better	28 (49%)	21 (54%)		
Worse	2 (3.5%)	0 (0%)		

Both groups experienced positive changes in EQ‐VAS scores from baseline to follow‐up, with mean increases of 5.2 points (SD: 9.6) in the all‐oral group and 6.2 points (SD: 9.2) in the standard group. Thresholds for a minimal clinically important difference (MCID) in EQ‐VAS are typically estimated at around 7–10 points in chronic respiratory diseases [21, 22], suggesting that the improvements observed here, although statistically meaningful, may not have reached the level usually considered clinically important. For EQ‐5D, an MCID of 0.03–0.05 is commonly applied [21, 22]; the domain‐level changes we observed are consistent with modest but tangible improvements in health‐related quality of life. However, a higher proportion of missing data in the standard regimen group (36.1% vs. 15.4%) limits the reliability of direct comparisons, as participants lost to follow‐up may have had different outcomes.

Improvements across the 5 EQ‐5D domains were observed in both groups. In the mobility, self‐care, and usual activities domains, a higher proportion of participants in the all‐oral group reported improvement compared to the standard group. For instance, improvements in mobility were reported by 53% of the all‐oral group versus 38% of the standard group. Similarly, gains in self‐care (49% vs. 41%) and usual activities (40% vs. 51%) were noted, although domain‐specific differences were modest.

Pain and discomfort improved in both groups. While a slightly greater proportion of the standard group reported improvements (54% vs. 44%), worsening pain was more frequent in the standard group (14% vs. 5.1%), suggesting less stable recovery. Symptoms of anxiety and depression also improved notably, with 49% of the all‐oral group and 54% of the standard group reporting better outcomes. Despite starting with a higher burden of symptoms, the all‐oral group achieved.

Substantial improvement by the end of follow‐up.

## 5 Discussion

This study compared the effectiveness, safety, and quality‐of‐life outcomes of two programmatic regimens for DR‐TB, implemented in semi‐concurrent cohorts under routine conditions. These findings arise from a comparative observational analysis of two cohorts delivered under programmatic conditions rather than from randomised or stepwise assignment. While both regimens demonstrated high effectiveness, the all‐oral regimen showed a non‐significant trend towards higher treatment success, lower mortality, fewer serious adverse events, and greater improvements in patient‐reported quality of life. These differences did not reach statistical significance, and the study was likely underpowered to detect modest effects. These results are consistent with the growing body of evidence supporting the shift towards all‐oral, bedaquiline‐ and linezolid‐containing regimens for DR‐TB [23, 24].

The all‐oral regimen achieved a 90.8% treatment success rate compared to 80.3% with the standard injectable‐containing regimen, largely due to higher treatment completion rates (29.3% vs. 9.8%). Between group differences in treatment success did not reach statistical significance, consistent with the limited size of our cohorts. Although the cure rate was slightly lower in the all‐oral group (61.5% vs. 70.5%), this is unlikely to reflect a true difference in clinical effectiveness and may instead relate to challenges in systematically confirming microbiological cure under programmatic conditions. Importantly, no cases of treatment failure were observed in either group, confirming the robustness of both regimens. These findings align with previous studies of shorter all‐oral DR‐TB regimens reporting success rates between 75% and 95%, depending on resistance patterns and settings [18, 25]. Although between‐group differences in our study were not statistically significant, the consistently higher treatment success observed with the all‐oral regimen suggests a favourable pattern in clinical effectiveness and reinforces current recommendations to replace injectable‐based regimens with safer, more tolerable all‐oral approaches. The improved outcomes observed with the all‐oral regimen are likely attributable in part to the inclusion of bedaquiline, which provides potent bactericidal activity sustained throughout the intensive and continuation phases of treatment. In contrast, the standard regimen relied on injectable aminoglycosides, protionamide, and high dose isoniazid, which are associated with more limited efficacy and greater toxicity. Evidence from multiple studies has demonstrated that regimens containing bedaquiline achieve higher treatment success and lower mortality compared with regimens without bedaquiline [26].

Twelve months after treatment completion, favourable outcomes were sustained in both groups. Mortality was lower in the all oral regimen group (7.5% vs. 16.4%), although this difference was not statistically significant. The higher mortality observed with the standard regimen may partly be attributable to the overall toxicity profile of injectable agents. Aminoglycosides are well recognised to cause nephrotoxicity, ototoxicity, and cardiac/electrolyte disturbances [27, 28]. The absence of TB recurrence in either group over 12 months of follow‐up is reassuring and suggests sustained effectiveness during this period, consistent with recent reports such as Rodriguez et al. (2025) [18].

Differences in adverse event profiles were consistent with known toxicity patterns. Anaemia occurred more often with the all‐oral regimen, whereas hepatotoxicity and QTcF prolongation were more frequent with the standard regimen, but several comparisons did not reach statistical significance. Anaemia was more frequent in the all‐oral group (46.2%), likely attributable to linezolid‐associated bone marrow suppression [29, 30], although most cases were mild or moderate and rarely led to treatment discontinuation. In contrast, hepatotoxicity and QTcF prolongation were significantly more common in the standard group (73.8% and 65.6%, respectively), consistent with the known toxicity of fluoroquinolones and injectable agents [31, 32, 33]. The higher rate of hepatotoxicity in the standard group is also likely explained by the inclusion of high‐dose isoniazid. While this strategy was adopted in the Bangladesh regimen and the STREAM trials, recent evidence shows that high‐dose isoniazid provides little benefit in strains carrying KatG mutations, the most common mechanism of isoniazid resistance. This raises concerns about both efficacy and safety, as the hepatotoxicity risk may outweigh any potential advantages [34]. While the overall burden of adverse events was similar between groups, the standard regimen was associated with more severe and life‐threatening events, contributing to a higher proportion of SAEs (19.7% vs. 12.3%). Although not statistically significant, this pattern suggests a potentially meaningful reduction in SAE risk with the all‐oral regimen. These findings reinforce the improved safety profile of all‐oral regimens and highlight the clinical disadvantages of injectable‐based treatments [24, 35].

Beyond clinical outcomes, differences in patient‐reported quality of life provide additional insights. Across EQ‐5D dimensions, participants receiving the all‐oral regimen reported more frequent improvements in mobility (53% vs. 38%). Improvements in usual activities (40% vs. 51%) and pain/discomfort (44% vs. 54%) were numerically higher in the standard regimen group. These variations may reflect differences in patient experiences of treatment burden and recovery, but the modest sample size and missing data limit definitive interpretation. Persistent concerns about linezolid‐associated toxicity, such as neuropathy and myelosuppression [6, 36], also remain important considerations in evaluating overall recovery.

The regimen selection for DR‐TB must balance effectiveness, safety, and patient experience. Our findings, showing fewer serious adverse events and greater quality‐of‐life improvements with the all‐oral regimen, align with WHO recommendations favouring all‐oral regimens, particularly in settings where injectables present substantial barriers [37, 38]. Importantly, the strong treatment adherence, low loss to follow‐up, and comprehensive long‐term follow‐up achieved under routine programmatic conditions in this study highlight the feasibility of delivering high‐quality DR‐TB care with all‐oral regimens.

Moving forward, patient‐centred care models that integrate pharmacovigilance, adherence support, and post‐TB care services will be essential. Generating real‐world evidence from diverse, resource‐limited settings will help ensure equitable and sustainable implementation of all‐oral regimens. Our findings confirm that all oral regimens can be successfully delivered under programmatic conditions in high‐burden settings and achieve high rates of favourable outcomes. Nevertheless, global guidance is now evolving. Both WHO and, more recently, CDC/ATS/IDSA/ERS guidelines recommend BPaL/BPaLM regimens as the preferred option for MDR/RR‐TB [39]. Outcomes with BPaL/BPaLM have been consistently superior to those of earlier all‐oral regimens [40]. Ultimately, scaling up safer, simpler, and better‐tolerated treatments has the potential to improve not only clinical outcomes but also the overall well‐being and long‐term recovery of individuals affected by DR‐TB.

## 6 Limitations

While this study provides valuable insights into the effectiveness, safety, and quality‐of‐life outcomes of two DR‐TB treatment regimens, several limitations should be acknowledged.

First, the non‐randomised observational design introduces the potential for selection bias. Because the cohorts were semi‐concurrent, secular changes in case mix, service delivery, or background risks may also have introduced temporal confounding. The absence of ‘not assessable’ outcomes strengthens confidence in the completeness of the data, although residual bias cannot be excluded. Although baseline characteristics were largely comparable, unmeasured confounders, such as differences in adherence, nutritional status, or healthcare access, may have influenced outcomes, and residual confounding cannot be excluded. Importantly, the study was not originally designed as a head‐to‐head comparison of regimens, and no formal sample size calculation was performed.

Second, the relatively small sample size (65 in the all‐oral group and 61 in the standard group) limits the precision of estimates and the ability to detect rare adverse events or subtle differences in effectiveness. In addition, as participants were drawn from selected DR‐TB treatment centres, generalisability to broader programmatic settings may be limited.

Third, the follow‐up period of 12 months post‐treatment may not fully capture late relapses, delayed toxicities, or long‐term post‐TB sequelae. Adverse events associated with key drugs such as linezolid (e.g., neuropathy, myelosuppression) and cardiac toxicities related to QTcF prolongation may emerge beyond the follow‐up window.

Fourth, missing data, particularly for patient‐reported outcomes, presents a potential source of bias. More than one‐third of participants in the standard regimen group lacked EQ‐VAS scores at 12 months, which could have led to overestimation of perceived quality of life and underestimation of ongoing disability or psychological burden.

Fifth, the study combined retrospective and prospective cohorts, with the all oral regimen data collected prospectively. Prospective follow‐up generally allows more complete and accurate capture of clinical events compared to retrospective record review, which may have introduced observer bias and contributed to differences in reporting between regimens. In addition, baseline disability assessments conducted retrospectively are subject to recall bias, which may have led to under‐ or over‐estimation of pre‐treatment functional limitations. Furthermore, causality assessment was not feasible for a subset of SAEs due to incomplete clinical documentation, particularly in the early implementation phase, which may have led to some uncertainty in the attribution of treatment‐related events.

Finally, this study did not evaluate the cost‐effectiveness, feasibility, acceptability, or scalability of implementing the all‐oral regimen. Although the all‐oral approach demonstrated better safety and quality‐of‐life outcomes, its higher cost, the need for pharmacovigilance infrastructure, and the management of linezolid‐associated toxicities require further investigation, particularly in resource‐limited settings.

To address these gaps, future studies should include larger and more diverse populations, extended follow‐up to capture late outcomes, and pragmatic evaluations of implementation challenges, costs, and health system readiness to support the broader scale‐up of safer, patient‐centred DR‐TB regimens.

## 7 Conclusions

This study demonstrates that high treatment completion, sustained follow‐up, and improvements in both functional and perceived health outcomes are achievable for rifampicin‐resistant TB under routine programmatic conditions. In this cohort, the all‐oral, bedaquiline and linezolid‐based regimen showed trends to fewer serious adverse events and more consistent functional recovery, although between‐group differences were not statistically significant.

These findings reinforce the global shift towards all‐oral DR‐TB treatment and highlight the need for continued innovation to deliver safer, shorter, and better‐tolerated regimens. As programmes scale up all‐oral approaches, ensuring robust pharmacovigilance and patient‐centred support systems will be critical to maximising treatment benefits, reducing long‐term morbidity, and strengthening health system resilience against drug‐resistant TB.

## Conflicts of Interest

Philipp du Cros works at the Burnet Institute, which is currently receiving funding from TB Alliance to support the roll‐out of the BPaL/M regimen. Philipp du Cros was on the steering committee for the TB‐PRACTECAL RCT.

## Supporting information


**Data S1:** tmi70041‐sup‐0001‐Supinfo.docx.
