# Supplementary material for: All‐Oral Shorter Treatment Regimens for Multidrug‐ and Rifampicin‐Resistant Tuberculosis: Evaluating Their Effectiveness, Safety, and Impact on the Quality of Life of Patients in Lao PDR
Source: Trop Med Int Health. 2025 Sep 25;30(12):1340–53. doi: 10.1111/tmi.70041 (PMC12675319; doi:10.1111/tmi.70041)
Supplement: Supplementary file 1 — Data S1: tmi70041‐sup‐0001‐Supinfo.docx. [file TMI-30-1340-s001.docx]

Supplementary Appendix

Table S1. MDR/RR-TB treatment regimens used in the study

| ***Comparator group*** | | | | | | | | | | ***Intervention group (ShORRT)*** | | | | | | | | | |
| --- | --- | --- | --- | --- | --- | --- | --- | --- | --- | --- | --- | --- | --- | --- | --- | --- | --- | --- | --- |
| Standard regimen | | | | | | | | | | All-oral regimen (for Fluoroquinolone sensitive) | | | | | | | | | |
| Months | M1 | M2 | M3 | M4 | M5 | M6 | M7 | M8 | M9 | Months | M1 | M2 | M3 | M4 | M5 | M6 | M7 | M8 | M9 |
| Clofazimine | X | X | X | X | X | X | X | X | X | Clofazimine | X | X | X | X | X | X | X | X | X |
| Pyrazinamide | X | X | X | X | X | X | X | X | X | Pyrazinamide | X | X | X | X | X | X | X | X | X |
| Moxifloxacin | X | X | X | X | X | X | X | X | X | Levofloxacin | X | X | X | X | X | X | X | X | X |
| Ethambutol | X | X | X | X | X | X | X | X | X | Bedaquiline | X | X | X | X | X | X |  |  |  |
| Amikacin | X | X | X | X |  |  |  |  |  | Linezolid | X | X |  |  |  |  |  |  |  |
| Isoniazid | X | X | X | X |  |  |  |  |  |  |  |  |  |  |  |  |  |  |  |
| Prothionamide | X | X | X | X |  |  |  |  |  |  |  |  |  |  |  |  |  |  |  |

Table S2. Dosing of medicines for adults

| **Drug** | **Weight group** | |
| --- | --- | --- |
|  | 30-50 kg | More than 50 kg |
| Bedaquiline (100 mg tablets) | 400 mg once daily for 2 weeks, then 200 mg 3 times per week afterwards | |
| Linezolid (600 mg tablets) | 600 mg once daily* | |
| Levofloxacin (250 mg or 500 mg tablets) | 750 mg | 1000 mg |
| Moxifloxacin (400 mg tablets) | 600 mg | 800 mg |
| Clofazimine (100 mg gel capsules) | 100 mg | 100 mg |
| Ethambutol (400 mg tablets) | 800 mg | 1200 mg |
| Pyrazinamide (500 mg tablets) | 1500 mg | 2000 mg |
| Isoniazid, high dose (300 mg tablets) | 400 mg | 600 mg |
| Prothionamide (250 mg tablets) | 500 mg | 750 mg |

**Linezolid dose is commonly reduced to 600 mg three times a week or 300 mg daily in patients with linezolid-induced peripheral neuropathy.*

Table S3. Schedule of examinations during treatment and follow-up phases of the study

|  | **Investigation/Observation** | **Baseline assessment & Screening** | **Treatment Phase (M=Month)** | | | | | | | | | **Follow-Up** | | |
| --- | --- | --- | --- | --- | --- | --- | --- | --- | --- | --- | --- | --- | --- | --- |
|  |  |  | **M _T_**  **1** | **M _T_**  **2** | **M _T_ 3** | **M _T_ 4** | **M _T_ 5** | **M _T_ 6** | **M _T_ 7** | **M _T_ 8** | **M_T_ 9/12** | **M_F_**  **6** | **M_F_ 12** |  |
| **Clinical evaluation** | Demographics, Medical History | X |  |  |  |  |  |  |  |  |  |  |  |  |
|  | Clinical Examination***** | X | X | X | X | X | X | X | X | X | X | X€ | X€ |  |
|  | Written informed consent | X |  |  |  |  |  |  |  |  |  |  |  |  |
|  | Treatment adherence |  | X | X | X | X | X | X | X | X | X |  |  |  |
|  | Concomitant treatment |  | X | X | X | X | X | X | X | X | X | X |  |  |
|  | Adverse events |  | X | X | X | X | X | X | X | X | X | X |  |  |
| **Bacteriology** | Sputum smear | X (2) | X | X | X | X (2) | X | X | X | X | X (2) | X | X |  |
|  | Sputum culture | X | X | X | X | X | X | X | X | X | X | X | X |  |
|  | DST (FQ/Injectables) | X |  |  |  |  |  |  |  |  |  | X**±** | X**±** |  |
| **Laboratory tests** | Haemoglobin/platelets count / White blood count | X | X# | X# | X# | X# | X# |  |  |  | X# |  |  |  |
|  | Serum creatinine (at baseline and if clinically indicated or ECG abnormalities) | X |  |  |  |  |  |  |  |  |  |  |  |  |
|  | Serum potassium (at baseline and if clinically indicated or ECG abnormalities) | X |  |  |  |  |  |  |  |  |  |  |  |  |
|  | Serum liver enzymes | X | X | X | X | X | X | X | X | X | X |  |  |  |
|  | Pregnancy test (female) | X |  |  |  |  |  |  |  |  |  |  |  |  |
|  | HIV and hepatitis test | X**µ** |  |  |  |  |  |  |  |  |  |  |  |  |
|  | TSH *(Note: for patients receiving Pto/Eto)* | X |  |  | X |  |  | X |  |  |  |  |  |  |
| **Other** | Chest X-ray**^ξ^** | X |  |  |  |  | X |  |  |  | X |  |  |  |
|  | ECG | X | X**β** | X | X | X | X | X | X | X | X |  |  |  |
|  | Visual acuity & BPNS*  *(Note: for patients receiving Lzd and high-dose INH/EMB)* | X | (X) | (X) | (X) | (X) | (X) | (X) | (X) | (X) | (X) |  |  |  |
|  | Audiometry *(Note: at baseline and monthly afterwards until end of treatment with injectable agents)* | X | X | X | X | X | X | X | X | X | X |  |  |  |
|  | Disability assessment | X |  |  |  |  |  |  |  |  | X | X | X |  |

**Patients were examined at least once a week for the first month of treatment and thereafter monthly throughout treatment. This task could be shifted to any health care staff trained and supported to interview and conduct basic examinations to detect adverse events. For patients taking linezolid or ethambutol, peripheral neuropathy screen and visual acuity and colour-blindness screening was recommended every month until linezolid and ethambutol are stopped.*

€ *Telephone contact was considered if* *patient transportation to the DR-TB treatment centre was not feasible.*

**±** *DST* *performed at baseline* *(with storage of the strain at -20c), after reversion during treatment, and if any culture is found positive, during the 12 months’ period after the end of the treatment (with storage of the strain of the recurrent episode for genotyping analysis), or after conversion.*

# *To be performed if the patient is taking* *linezolid.*

µ *Hepatitis B (HepBs Ag) and hepatitis C (HCV Ab) tests.*

Β *Baseline ECG + potassium was obtained and additional ECGs conducted at week 1 and 2 after starting treatment and thereafter monthly throughout treatment. ECG was repeated as necessary in case of clinical suspicion of heart rhythm and conduction disturbances, or other clinical signs (e.g. dehydration and electrolyte misbalance).*

*F: The proposed follow-up was 2 visits every 6 months during one year. Patients werre advised to present at health facility as soon as they experienced TB symptoms.*

**ξ** *Chest X-ray could be repeated during follow-up in case of suspicion of recurrence. Extent of disease was assessed based on the degree of radiographic involvement observed on chest X-ray at baseline.*
